# Steps Toward a Philosophy for Mathematicians

**DOI:** 10.1007/s00283-022-10190-1

**Published:** 2022-08-10

**Authors:** Jens Erik Fenstad

**Affiliations:** University of Oslo, Oslo, Norway

A *philosophy of mathematics* is not necessarily a philosophy for mathematicians. This was forcefully argued by Ian Hacking in his recent book *Why Is There Philosophy of Mathematics at All?* [19], in which he expresses a deep skepticism of almost all there is of current philosophy of mathematics and reports at length on actual mathematical practice, on the nature of proofs, and on the variety of applications as seen within the mathematical community. His discussion contains deep and important insights, but it seems fair to say that no general foundation for the working mathematician emerges in a convincing way from his account.

There are many points of contact between Hacking’s book and the approach that we developed in our 2018 book *Structures and Algorithms* [16]. We believe that it is possible to extract from that book a reasonably complete philosophy for the working mathematician. This is what we shall attempt to do in this paper, using Thoralf Skolem as our guide.

But before starting on this task, we should note that there is currently much work being done in what is called the philosophy of mathematical practice; for a brief overview of this field, see the 2008 collection [25] edited by Paolo Mancosu. There is much common ground between the philosophy of mathematical practice as discussed in [25] and the philosophy for mathematicians that is the topic of this work. But there is an important difference: the former attempts to analyze what mathematicians do, whereas our interest is to understand what they should be doing.

## Skolem 1920

One hundred years ago, the Norwegian mathematician Thoralf Albert Skolem published the first of a series of remarkable papers on logic and the foundations of mathematics [33]. That 1920 paper, modestly announced as some “logical-combinatorial investigations”^1^ on the satisfiability and provability of mathematical propositions, introduced new technical tools into logic such as what are now known as Skolem functions and Skolem normal forms. The main technical result was an extension of a 1915 result of Leopold Löwenheim [23] on the existence of countable models for finite and countably infinite sets of consistent first-order formulas. That result, known as the extended Löwenheim–Skolem theorem, was a basis for Skolem’s next paper [34], published in 1922, titled “Some remarks on axiomatized set theory.”^2^ In that paper, Skolem first made some important improvements to the Zermelo axiomatization before using the Löwenheim–Skolem theorem to prove the existence of countable models for every first-order set of axioms for set theory.

In 1923, he published a paper rich in content on the “recursive mode of thought” (rekurrierende Denkweise) as a basis for elementary arithmetic. This paper has been seen as a first step toward the development of recursive mathematics. It is also possible to see the 1923 paper as a starting point for a line of investigation, continued in [36], on a bottom-up combinatorial approach to provability in first-order logic. Finally, in 1934, he published a paper [39] titled “On the impossibility of a complete characterization of the sequence of integers by means of a finite or countably infinite axiom system with exclusively integer variables.”^3^ This was a remarkable result showing that not only set theory but also Peano arithmetic necessarily admits nonstandard models.

## Skolem Pre-1920

In order to understand what Skolem achieved during the 1920s, one must have a proper understanding of what he did during the previous decade. To highlight just one example, his great paper on the “recursive mode of thought” cannot be understood without taking into account his experiences as a young mathematician in Göttingen during the First World War. We quote from [15, pp. 62–63]

“Es kommt doch auch bei der Begründung der Mathematik auf die Sache und nicht auf die Bezeichnung an.” The words are Skolem’s and occur towards the end of his 1923 paper on the *Begründung der elementaren Arithmetik durch die rekurrierende Denkweise.*^4^ Thoralf Skolem, who wrote this paper in 1919, had at that time become acquainted with the approach to elementary arithmetic in the *Principia Mathematica* of Russell and Whitehead, and he was not at all convinced. Natural numbers and their fundamental properties need not—and should not—be justified through an exercise in abstract logic. This seems to be a strongly held belief of Skolem, firmly rooted in his early education and work experiences. By 1919 he had already been exposed to a wide range of problems and projects in both pure and applied mathematics. His master thesis in mathematics from 1913 [31] was a profound study of the algebraic logic of Peirce and Schröder. His first employment was as a student assistant to the well-known Norwegian physicist K. Birkeland in the years 1909 to 1914. His special assignment was to help with the mathematics of several geophysical phenomena. In 1917 he published his first major mathematical paper *Untersuchungen über einige Klassen kombinatorischer Probleme.*^5^ It is therefore not surprising that he was not at all convinced when he in Göttingen in the winter 1915–1916 first learned about the Russell and Whitehead approach to elementary arithmetic in the *Principia Mathematica* [46]*.* We get the impression that Skolem through his work in algebra, combinatorics and geophysics “knew” what numbers are, and that his 1923 paper can be seen as a protest against the approach of Russell and Whitehead. In this paper he takes the first steps towards a more “correct” approach by developing what has later become known as primitive recursive arithmetic, and we would argue that to choose the “rekurrirende Denkweise” as a foundation for “real” mathematics was almost to be expected from an expert in combinatorial analysis. He develops his theory within a free variable logic formalism, but he is not entirely satisfied. Too much of “die Bezeichnung” of Russell and Whitehead remains, and he promises a further paper on the real “Sache,” which will strictly follow the views of Kronecker that “eine mathematische Bestimmung dann und nur dann eine wirkliche Bestimmung ist, wenn sie mit Hilfe endlich vieler Versuche zum Ziele führt.”^6^ But the promised paper never appeared.

Neither Kronecker nor Skolem tells us what a number exactly is. But an answer to this problem is, however, only part of the challenge. The world of mathematics contains, as we noted above, in addition to the “discrete” objects of arithmetic, also the “continuous” objects of geometry. Is Russell’s logic and Zermelo’s axioms for Cantorian set theory an answer to the question of existence? Neither Kronecker nor Skolem would agree. Mathematics is more than an axiomatic exercise in logic and abstract set theory. In a popular lecture given at the Christian Michelsen Institute in Bergen in 1932 [38] Skolem says:

If one works within a completely formalized mathematics, based on a finite number of precisely stated axioms, there is nothing to discuss but questions of consistency and ease of manipulation. But in ordinary mathematical practice, e.g. in the usual studies on continua which are never given by a set of specified formal construction principles, the axiom of choice is, in my opinion, definitely undesirable – a kind of scientific fraud.^7^

Leaving the question of “scientific fraud” aside, a challenge to “ordinary mathematical practice” remains, to explain what kind of objects numbers and geometric objects are.

This ends our quotation from [15]. We shall return to the promised Skolem foundation of mathematics in the last section of this paper. But first some remarks on Skolem as a working logician—what he did and what he missed.

## Skolem the Founder

What Skolem did in [40] is well summarized by Hao Wang in [45]. We quote here from the updates in the Fenstad–Wang article on Skolem in [17, p. 134]:

A comprehensive unifying theme is the nature of quantifiers (with infinite ranges):

1. The Skolem functions and countable models as a general method of analyzing quantifiers both in pure logic and in axiomatic theories.
2. The founding of recursive arithmetic as a method of developing mathematics on a quantifier-free basis.
3. The invention and powerful applications of the decision method of eliminating quantifiers for axiomatic theories.
4. Various results on the decision and reduction problems of quantification theories (e.g. the Skolem normal form).
5. The classic explication of Zermelo’s concept of “definite property” by the notation of quantification theory.
6. The discovery and emphasis on non-standard models of axiomatic number theory and set theory.

Indeed, as Wang concludes in his survey, Skolem “participated in the founding of these subjects by introducing initially several of the basic ideas in their concrete and naked form” [17, p. 135].

Skolem’s positive contributions are truly impressive. But great men may sometimes have a “blind eye.” We have listed a series of results of what he saw. Let us balance this by one rather important example of what he did not see: the case of infinitesimals. We quote a few paragraphs from [15, pp. 58–60]:

A secure logical foundation for analysis emerged only in the 1870s through the work of Cantor, Dedekind and Weierstrass. And the new foundation had no room for infinitesimals. This was strongly expressed by A. Fraenkel in his well known text on Mengenlehre: “Bei dieser Probe hat aber das Unendlichkleine restlos versagt.”^8^ In a certain sense, set theory and its use in the foundation of analysis marked a victory for the discrete over the continuous: In the beginning there is the empty set—the rest, including the geometric continuum, is an exercise in set theoretic constructions.

The standard view on analysis was firmly seconded by Skolem. In a lecture to a Scandinavian congress in 1929 [37] he argued against using infinitesimals as a foundation:

We know that such entities can be introduced—they can be exhibited in so-called non-Archimedean number systems—but it is not possible to construct a calculus of infinitesimals on such a foundation.^9^

There may indeed be entities in the mathematical universe that exhibit the “defining” properties of infinitesimals, but the question of interest to us is if infinitesimals exist in an (elementary) extension of the real number system, and if there is enough room on the “real” geometric line to faithfully represent the extended number system. This is what Skolem denies in his 1929 lecture.

But do not always listen to what great men say. In 1934 Skolem constructed his non-standard extension of the natural number system,^10^ i.e. he constructed an “object” *N as a proper elementary extension of the natural number system **N**, where “elementary extension” means that all the basic rules of arithmetic are preserved in the extension from **N** to *N. As a proper extension *N must contain at least one infinitely large number, hence the inverse of any infinitely large number in *N will be a true infinitesimal. This was observed by Abraham Robinson in 1960:

In the fall of 1960 it occurred to me that the concepts and methods of contemporary Mathematical Logic are capable of providing a suitable framework for the development of the Differential and Integral Calculus by means of infinitely small and infinitely large numbers.^11^

Starting from the infinitely large numbers and the infinitesimals, Robinson was led to a richer set of points on the geometric line, usually called the hyper-reals and denoted by *R. We recall that *R is, in the technical language of logic, a proper (and saturated) elementary extension of **R**, containing both infinitesimals and their inverses, the infinitely large numbers; see in addition to Robinson also Keisler^12^ and Fenstad.^13^ The construction of the coordinate field *R is from our point of view primarily a method of constructing new points on the geometric line. The coordinate set *R has strong closure properties and we may, similarly to the standard approach, “forget” the ambient geometric space and choose one version of *R as the extended geometric line. But this is not, we would insist, correct. The set of “coordinates” *R is not the geometric object, but only one method of “naming” points in the continuum. We are therefore at liberty to create different “point-sets,” or more correctly, different coordinate structures, on the geometric line for different purposes.

So much for our quotation from [15]. We add that Robinson was careful to acknowledge his debts to Skolem’s work on nonstandard models of arithmetic. Skolem died in 1963, and it is highly doubtful whether he ever learned of the new infinitesimal analysis. We can therefore not know whether he would have changed his view from 1929 and admitted that there is room for infinitesimals on the geometric line.

That is history past. Today, nonstandard analysis is a thriving branch of mathematics. The tension between the discrete and the continuous has always been a central theme in mathematics. Infinitesimals can be seen as a bridge between the two; see [42] and the recent survey [18] by Goldbring and Walsh. We shall return to this “tension” in the next section.

## Skolem the Skeptic

Toward the end of the nineteenth century, there was a development toward a unified approach to mathematics. A driving force behind this development was the need to understand the nature of “real” numbers. Mathematicians had learned to live with the tension between counting and the discrete on the one hand and geometry and the continuous on the other. This tension has a long history. We may mention early sources such as the *Liber Abbaci* by Fibonacci from the early thirteenth century on the discrete and the book *A Commentary on the First Book of Euclid’s Elements* by Proclus from the fifth century on the continuous. For a long period, the two views lived peacefully side by side, with professional mathematicians being happy to apply their new tools both to nature and to problems internal to the formalism, Euler being an excellent example. But during the nineteenth century, there was a growing unease inside the mathematical tribe concerning the “correct” understanding of how an arithmetic approach to the geometric continuum could be possible in a consistent way.

One way of connecting the two is through the act of measurement. Measurements create in the continuum points and intervals, hence also parts of or fractions of intervals. But do the points we create through this process exhaust the continuum? Is the geometric continuum a set of points each labeled by a unique “number”?

There were at the time two possible candidates for a foundation of mathematics, one being logic and Russell’s theory of types and a second being Cantor’s set theory. Many mathematicians nodded politely in the direction of Russell’s theory of types but turned to Cantor for their daily work. A notable example is G. H. Hardy. One factor in favor of Cantor was the Dedekind analysis of numbers and the follow-up construction of the reals, which reached a high point in the later axiomatization of set theory by Zermelo. This seemed to be the way forward, but at least one young mathematician was at the time not convinced.

That man was Skolem. We saw above in the section “Skolem Pre-1920” how he reacted to the Russell–Whitehead-type theoretical analysis of natural numbers, and also his skepticism toward any first-order axiomatization of set theory based on his proof of the existence of countable models for first-order theories. For Skolem the skeptic, it was structures and algorithms that were of importance—axioms are at most a tool, not a foundation.

In a certain sense, set theory resolved the tension between the discrete and the continuous in favor of the discrete. But today we have seen how geometry has fought back. The story is a bit complicated, but it needs to be told. Our starting point is a lecture by Per Martin-Löf from 1983, “Constructive Mathematics and Computer Programming” [26]. He starts from the fact that computing and algorithms have become of increasing importance in mathematics. But he further notes that if one wants to move from computer science (the technology of the computing device) to computing science (the algorithms), there is a need for an appropriate language that reflects this shift in structural understanding. He then observes that current set theory is not a suitable formalism for this purpose and proposes to build on his constructive theory of types. We see an echo of Russell and his theory of types in this proposal, but Martin-Löf moves far beyond that.

The task set by Martin-Löf in using his constructive type theory as a programming language was not at all trivial; in particular, great care has to be taken in the treatment of the so-called identity types, but he succeeded. A first success was several important advances in the construction of proof assistant systems. But that is not the end of the story.

Martin-Löf’s analysis was dominated by syntactic and proof-theoretic concerns. But questions about “identity” were also present in other parts of mathematics; see the recent “Modeling Homotopy Theories” [5]. It turned out that these were two sides of the same concern: a certain class of homotopy type structures was seen to provide a “true” semantic interpretation of the syntax of the Martin-Löf theory [4]. This important insight has led to an extensive development, and as expected, it is now the semantics, that is, the geometric structure, that occupies the central role. The discrete has disappeared in favor of *synthetic spaces*, an approach to geometry based on “abstract shapes” not initially built out of points; we quote a few words from an introductory survey to this topic by Shulman [30]:

if all objects in mathematics come naturally with spatial structure, then it is perverse to insist on defining them first in terms of bare sets, as is the official foundational position of most mathematicians, and only *later* equipping them with spatial structure. Instead, we can replace set theory with a different formal system whose basic objects are spaces.

With HOTT, which is now the standard acronym for the new *homotopy type theory*, we have a genuine geometric approach to mathematics; see the Bernays Lectures by Voevodsky [44] and the Skolem Lecture by Awodey in [3].

Let us at this point recall a comment made earlier. We had taken note of the tension between the discrete and the continuous and how the infinitesimals in a certain sense act as a bridge between the two. We had already, in the early 1980s, suggested that the geometric real line is not a point set, see [12], and showed how this point of view had offered richer possibilities to model natural phenomena; see as an example the paper [1] on singular perturbations of differential operators by Albeverio et al. But a synthetic theory of “abstract shapes” was not part of our agenda.

Skolem did not live to see these developments. But if he had, how would he have responded? This is a question that we, of course, cannot answer. We know that Skolem was interested in structures and not in axioms. As one example, we know that he admired Cantor’s ordinals, not as a foundation, but as a new and highly interesting mathematical structure; see [17, p. 176]. It is possible that he would be equally fascinated by the “abstract shapes” of synthetic geometry, but that does not mean that he would necessarily subscribe to a HOTT foundation for mathematics.

Set theory and homotopy type theory (HOTT) are both universal axiomatic theories of mathematics—they have strength and beauty. But we note that the “grand view” of mathematics toward which these developments aim stands in stark contrast to Skolem’s skepticism and his insistence on the primacy of specific structures and doable algorithms.

## Steps Toward a Possible “Skolem Foundation”

The words “foundation” (*Begründung*), and “foundational questions” (*Grundlagenfragen*) occur frequently in the titles of Skolem’s papers. But to what extent did Skolem have a complete and coherent view of the foundation of mathematics? This was a question discussed at length in the concluding section on Skolem in [17]. We have in the present survey emphasized that the young Skolem was a mathematician interested in structures and algorithms, working on the algebra of logic and on combinatorics. We also observed that despite the fact that Skolem was an expert in using the axiomatic method, he did not believe in an axiomatic characterization of basic concepts such as *numbers* and *sets*. Concerning numbers, this was clearly expressed in the great 1923 paper on the “recursive mode of thought” (*rekurrirende Denkweise*). Concerning sets and the continuous, we have his opinion from the popular 1932 lecture that “in the usual studies on continua” they are “never given by a set of specified formal construction principles.” Skolem the skeptic always knew what he did not like. But we would also like to know whether there is a positive side. Could there be a “Skolem philosophy” faithfully representing his views on the “real” nature of numbers and sets?

An answer to this question presupposes that we have a “correct” understanding of what knowledge is. At this point, we enter highly controversial ground. Our interest is knowledge and mathematics. Do we have to choose between such extremes as the *formalism* of Abraham Robinson and the *platonism* of Kurt Gödel concerning the existence of abstract objects? Or is the *constructive* approach of Brouwer preferable? We have shown elsewhere that there is a middle ground, and that is *culture;* see [17].

The reader will find an extended discussion of the cultural approach in the book *Structures and Algorithms, Mathematics and the Nature of Knowledge* [16]. Here we recall a few points from the analysis in the postscript to Chapter 9 of that book. We want to understand the general nature of knowledge, of what we “see” and what we “do.” Structures and algorithms, seen as abstract cultural objects, are essential for this understanding. Language and other formalisms, in particular the language of mathematics, serve as a link between the two. There is, however, no mathematics without numbers, either directly as objects or more generally as tools. Understanding knowledge thus means to know what numbers are.

Numbers are not objects in space and time; it thus seems necessary to acknowledge, as noted above, the existence of abstract objects in addition to physical objects and states of (individual) minds. The belief in abstract mathematical objects is widespread within the mathematical community; see the book *Why Belief Matters: Reflections on the Nature of Science*, by E. Brian Davies [6]. Davies is in no doubt about what mathematics is: “Mathematics is an aspect of human culture, just as are language, law, music, and architecture” [6, p. 101]. This is a strong and unqualified expression of how mathematics is grounded in human culture, a view that was severely criticized by Husserl: there are mathematical objects, but they are neither social or cultural objects, because such objects are bound to time and place. In [16], we tried to strengthen the cultural view against this criticism with arguments drawn from both neuroscience and evolutionary theory. These arguments seem to be strengthened by recent results that show, contrary to the common belief that large evolutionary change requires thousands or millions of years, that the human genome can change significantly in as little as a generation; see the review in [27]. Thus, what is “seen” can leave an imprint on the human genome and hence become independent of time and place.

As mentioned in [16, p. 128], the approach in “A Second Philosophy of Arithmetic,” a recent paper by Penelope Maddy, “seems to have many points of contact with the views developed” in the postscript to [15]. “To summarize, then, the Second Philosopher concludes that much of the world displays a familiar abstract template—a domain, properties and relations, dependencies ….” [24, p. 225]. This view also expresses a widespread attitude in the mathematical community: first structure, then syntax and rules. A forceful expression of this view is found in Rawls’s report on the celebrated Sacks–Dreben slugfest in 1993. Both are well-known Harvard professors, Burton Dreben in philosophy, Gerald Sacks in mathematics. Sacks argued that what is important for logic is structure, not syntax. “In learning mathematics, we become aware of and experience structure. This is what mathematical experience is ….” Sacks granted that syntax is simpler and that it therefore makes sense that we begin teaching logic with syntax; see [28, p. 420].

How are abstract computational structures and physical calculations connected? Let us first make a few remarks on language and brain following [14]; for a current update, see the 2019 survey on *Language and the Brain* in the journal *Science*.^14^ We assume that what we “see”—objects and properties—have through an evolutionary process given rise to some rudimentary form of “universal” protosyntax (such as the decomposition of a whole message S into an NP + VP structure), which in turn has left an imprint on the human genome. Thus “universal grammar” is grounded in culture. We would like to believe that the situation with numbers and calculations is similar to the language case. As noted above, the speed of evolutionary processes seems to be much faster than we originally believed—from millions of years, we now move to generations or at most some thousand years. This makes it possible that the abstraction of numbers as cultural objects from rudimentary computational practice, as described in [22], has in turn left an imprint on the human genome; see [16, chapter 9]. Thus, similarly to the case of language, we have a “universal arithmetic” grounded in culture. What is “imprinted” and what are the exact neural mechanisms are still unknown, but perhaps some clues can be found in the earlier models of Dehaene and Changeux [9]; see also [8] and [7]. If this is a correct line of reasoning, we can argue contra Husserl that numbers, invented by man, have become independent of time and place. It is of interest to note that the recent development of the Gödel–Husserl view by Richard Tieszen—see in particular his 2015 paper [43]—seems to narrow the gap between numbers seen as abstract cultural objects and numbers seen as objects in the particular “World of Arithmetic” of phenomenology. But the gap remains.

We note that there also seems to be an implicit cultural foundation for Maddy’s “Second Philosophy.” The approach developed in *Structures and Algorithms* [16] is more directly cultural, and believing in numbers as (abstract) objects, we are probably “thin realists” in her view. The cultural approach needs numbers as objects. No one who has read C. P. Snow on Hardy’s visit to Ramanujan in hospital will doubt this:

Hardy had gone out to Putney by taxi, as usual his chosen method of conveyance. He went into the room where Ramanujan was lying. Hardy, always inept about introducing a conversation, said, probably without a greeting, and certainly as his first remark: “I thought the number of my taxi was 1729. It seemed to me a rather dull number.” To which Ramanujan replied: “No, Hardy! It is a very interesting number. It is the smallest number expressible as the sum of two cubes in two different ways” [41, foreword].

There are two lessons to be learned. First, numbers exist. Second, there is a difference between knowing a number and knowing what properties a number has. In this respect, abstract and physical objects are similar; “invention” and “discovery” are not necessarily contradictory. Numbers are cultural objects invented by man. But no man knows what properties there are to be discovered.

So far, we have presented the cultural approach to mathematics found in *Structures and Algorithms*. But could this approach also serve as a “Skolem foundation” for mathematics? Are Skolem’s views on the foundation of arithmetic from 1923 and his remark “on the usual studies of continua” in the popular 1932 lecture sufficient to establish this claim? What we can say so far is that a cultural foundation is not inconsistent with his many remarks on foundational issues.

In our search for a Skolem foundation we leave for a moment the rarified world of mathematics pure and abstract and turn to mathematics in practice. A first observation is that mathematical models in science and technology are usually “small.” They can, if desired, be described inside a finite part of the countable hierarchy over some “basic structure.” And the basic elements for such structures are numbers, lines, and shapes, and even infinitesimals, all existing as cultural objects in the “mind of the species” (our common cultural space). And we seem to have great freedom of choice at every stage of any modeling process: all *structures* need not live in the same universe; the *language and logic* (be it classical, constructive, type-theoretic, etc.) used to describe the structures we see should be adapted to the problem at hand; *algorithms,* being equally an art form and a well-defined science, can come from anywhere. For a related view, see Hosack [20]. His *deductive pluralism* has many points of contact with the view expressed here, but our emphasis on the primacy of structures seems to be a better starting point for a philosophy of mathematical practice than his deductive pluralism.

At this point, it is appropriate to add some remarks on what is needed for “efficient” computing in the emerging age of “big data”; see the Tukey Centennial Lecture by D. Donoho from 2015 [10] and the ICM lecture on the *Mathematics of Machine Learning* by S. Arora from 2018 [2]. Traditionally, efficient computing has been restricted either to “small” discrete systems (combinatorics) or continuous systems (standard numerical analysis); see Chapters 1 and 3 in *Structures and Algorithms.* New methods, usually referred to as “machine learning,” are now beginning to bridge the gap between the two cases; see [10]. It remains to be seen whether hyperfinite combinatorics will find a place in this picture; see [42]. Finally, note that computing in practice pays little attention to the general theory of computability, except when one is trying to prove that no algorithm exists, such as in the case of Hilbert’s tenth problem; see the discussion in [13].

We need to add a few words on what we mean by “basic structure.” We insist that basic structure is not a set-theoretic construction, but more like a “type” in the HOTT approach, i.e., something “seen” and not constructed. In our view, basic structures are cultural objects, not models of some axiomatic system for set theory. There are indeed several critical observations to a possible set-theoretic approach to “basic structure.” First, and perhaps most important, any set-theoretic formalization preserves at most the proof structure, not what was “seen” in the basic structure; understanding the “true nature” of numbers and the geometry of abstract shapes is a key example of this crucial difference between “proofs” and “insights.” And of equal importance, internal problems with a set-theoretic coordinate structure for some basic geometric structure are not necessarily “problems” concerning the given “basic structure,” the continuum hypothesis being a notorious example. Finally, note that mathematical practice does not need a “total formalization,” except in the case in which one also wants to add a general computer proof assistant to one’s theory.

It is also of some interest to observe that our theory of “small worlds” fits equally well the way physicists work and behave in their daily lives. The majority of this tribe live and experiment within many different “small worlds” without the need for any form of overarching unified theory; see, in particular, Chapter 4 of *Structures and Algorithms*. There are indeed some, Freeman Dyson is one example, who believe that there is no unified theory of quantum effects (points) and general relativity (geometry); see [11]. Thus, a theory of “small worlds” is emerging as a valid and well-argued approach to both mathematics and the natural sciences.

But would this also be a “Skolem philosophy” of mathematics? In order to answer this question, we need to recall the distinction we made in the introduction between a “philosophy of mathematics” and a “philosophy *for* mathematicians.” The former designates today an independent intellectual activity of great beauty and complexity. It is, however, neither a specialty inside mathematics nor a necessary foundation for doing mathematics. For the latter, a philosophy *for* mathematicians, we return to Skolem. His pre-1920 work experiences, his 1923 paper, and his 1932 remarks seem to justify that a foundation based on a cultural understanding of mathematical objects, together with an insistence on the primacy of structures and algorithms and a “small world” approach to mathematical modeling, is what is needed for the working mathematician.

**Postscript:** Jens Erik Fenstad passed away in April 2020, shortly before his 85th birthday, as an early victim of the coronavirus pandemic. He had worked extensively on this article in his final year, including several long discussions with me. After falling ill, Jens conveyed to his wife that he considered the article ready for publication and requested that I try to find a suitable venue for it. He would no doubt have been very pleased about its publication in the Mathematical Intelligencer. A short obituary (in Norwegian) can be found at https://www.mn.uio.no/math/om/aktuelt/aktuelle-saker/2020/jens-erik-fenstad.html.

—Øystein Linnebo, Professor of Philosophy, IFIKK, University of Oslo

## Footnotes

1 “Logisch-kombinatorische Untersuchungen.”

2 “Einige Bemerkungen zur axiomatischen Begründung der Mengenlehre.”

3 “Über die Nichtcharakterisierbarkeit der Zahlenreihe mittels endlich oder abzählbar unendlich vieler Aussagen mit ausschliesslich Zahlenvariablen.”

4 Skolem, “Begründung Der Elementären Arithmetik Durch Die Rekurrierende Denkweise Ohne Anwendung Scheinbarer Veränderlichen Mit Unendlichem Ausdehnungsbereich” [35]. The German text is translated as “Yet, even in providing a foundation for mathematics it is the substance that is important, not the notation” in van Heijenoort, *From Frege to Gödel*, 333. I am not happy with the translation “substance” of the word “Sache”; I would argue that “Sache” in this context refers to what there is.

5 Skolem, “Untersuchungen über Einige Klassen Kombinatorischer Probleme.” Oslo [32].

6 This is translated as “… a mathematical definition is a genuine definition if and only if it leads to the goal by means of a finite number of trials” in van Heijenoort, *From Frege to Gödel,* p. 333.

7 The original text is in Norwegian; see Skolem*, Undersøkelser over Potensrester Og over Logisk Karakterisering Av Tallrekken*: “Vil man gå over til en konsekvent formalistisk matematikk, basert på et endelig antall eksakt formulerte aksiomer, så er der intet annet å innvende, enn at der kan være spørsmål om motsigelsesfrihet og hensiktsmessighet o. l. Men innenfor den matematiske praksis, slik den vanligvis drives med kontinua, som slett ikke er gitt i kraft av de og de bestemte oppregnede konstruksjonsprinsipper, er utvalgsaksiomet efter min mening avgjort en uting—en slags videnskabelig svindel.”

8 “In this test, however, the infinitely small has completely failed.” Fraenkel, *Einleitung in die Mengenlehre*, p. 116. The test referred to is how to prove a mean value theorem for arbitrary intervals, including infinitesimal ones; see section 6.1, on the Klein–Fraenkel criterion, in Kanovei, Katz, and Mormann, “Tools, Objects, and Chimeras.”

9 Skolem, “Über die Grundlagendiskussionen in der Mathematik.”

10 Skolem, “Über die Nichtcharakterisierbarkeit der Zahlenreihe mittels endlich oder abzählar unendlich vieler Aussagen mit ausschliesslich Zahlenvariablen.”

11 Robinson, *Nonstandard Analysis* [29].

12 Keisler, “The Hyperreal Line” [21].

13 Fenstad, “Nonstandard Analysis Relevant for the Philosophy of Mathematics?”; and Fenstad, “+The Discrete and Continuous in Mathematics and the Natural Sciences.”

14 *Science* 366:6461 (2019), 48–66.
